# Eye-Strain in Health and Disease, with Special Reference to the Amelioration or Cure of Chronic Nervous Derangements without the Aid of Drugs

**Published:** 1898-09

**Authors:** 


Eye-Strain in Health and Disease, with special reference to the amelioration or cure of Chronic Nervous Derangements without the Aid of Drugs, by Ambrose L. Ranney, A. M., M. D., author of "Lectures on Nervous Diseases," "The Applied Anatomy of the Nervous System," etc., etc.; late Professor of Nervous Diseases in the Medical Department of the University of Vermont, and of the Anatomy of the Nervous System in the New York Post-Graduate Medical School, etc. Illustrated with 38 wood cuts. One volume, royal octavo, pp. viii-321. Extra cloth, beveled edges, $2 net. The F. A. Davis Company, publishers, 1914 and 1916 Cherry street, Philadelphia; 117 West Forty-second street, New York; 9 Lakeside Building, Chicago.

In this volume Dr. Ranney gives the result of his experience and observation in the production of various nervous symtoms and phenomena by a lack of normal power and balance among the orbital muscles; and the possibilities of effecting a cure of many functional disorders, including headache, chorea, epilepsy, etc., by accurately determining the anomalies and correcting them by operation on the tendon of the affected muscle.

That there is much in this theory of eye-strain being the cause of various nervous ailments we think there can be no reasonable doubt, but whether further investigation will bear out the enthusiastic conclusions of Dr. Ranney, or even go further, remains an open question. The book is well worth careful study, whether one accepts all of the author's views or not. S. E. H.

